# Supplementary material for: Rapid and Sensitive Electrochemical Assay of Cefditoren with MWCNT/Chitosan NCs/Fe2O3 as a Nanosensor
Source: Micromachines (Basel). 2022 Aug 19;13(8):1348. doi: 10.3390/mi13081348 (PMC9412661; doi:10.3390/mi13081348)
Supplement: Supplementary file 1 [file micromachines-13-01348-s001.zip › micromachines-1852867-supplementary.pdf]

Rapid and Sensitive Electrochemical Assay of Cefditoren with MWCNT/Chitosan NCs/Fe_2O_3 as a Nanosensor

Nida Aydogdu [1,2], Goksu Ozcelikay [2], Sibel A. Ozkan [2,*]

[1] Department of Analytical Chemistry, Faculty of Pharmacy, Afyonkarahisar Health Sciences University, 03218 Afyonkarahisar, Turkey
[2] Department of Analytical Chemistry, Faculty of Pharmacy, Ankara University, 06560 Ankara, Turkey
* Correspondence: sibel.ozkan@pharmacy.ankara.edu.tr

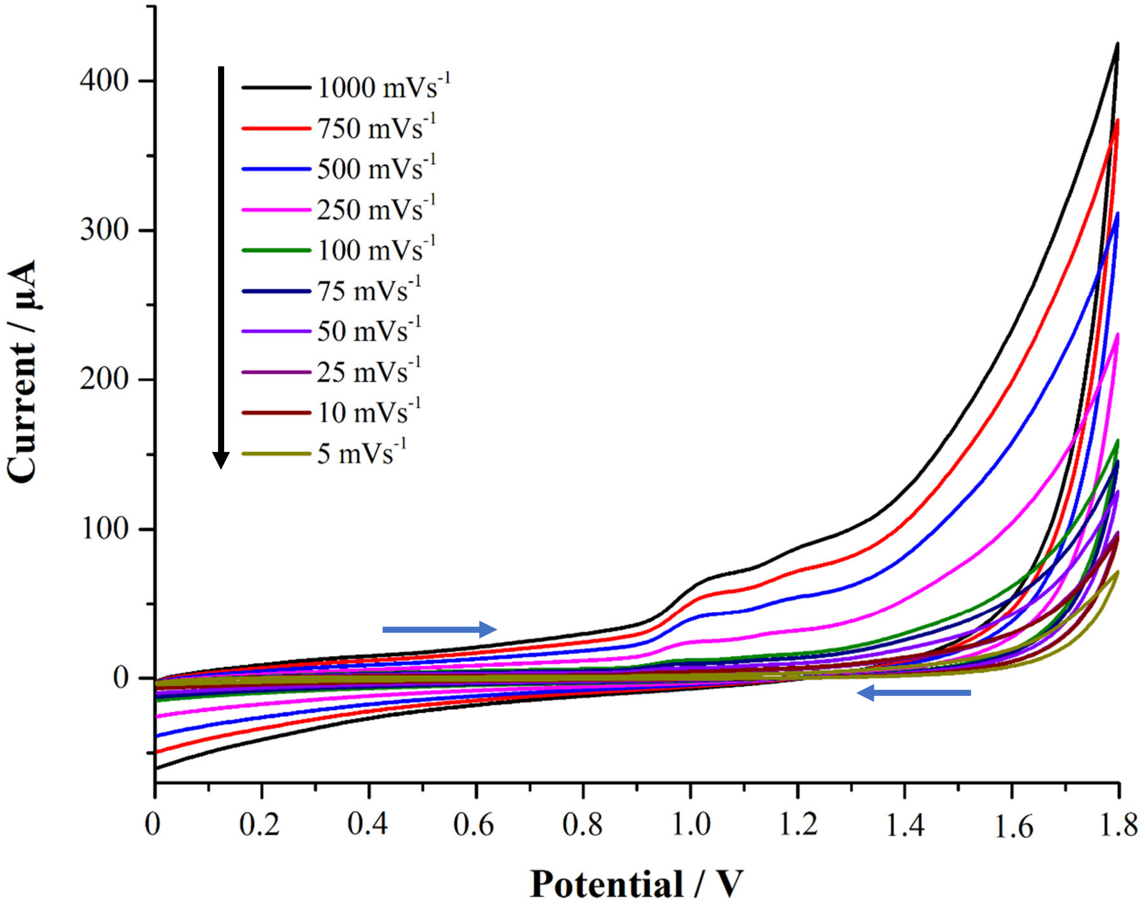

Figure S1. Cylic voltammograms of different scan rates of 1 × 10^{-4} M CFT solutions in 0.1 M H_2SO_4 by MWCNT/Chitosan NCs/Fe_2O_3/GCE (CV parameters: 0.2 V potential condition, 5 s time condition, 5 s equilibration time, 0.0 V potential begin, 0.005 V potential step)

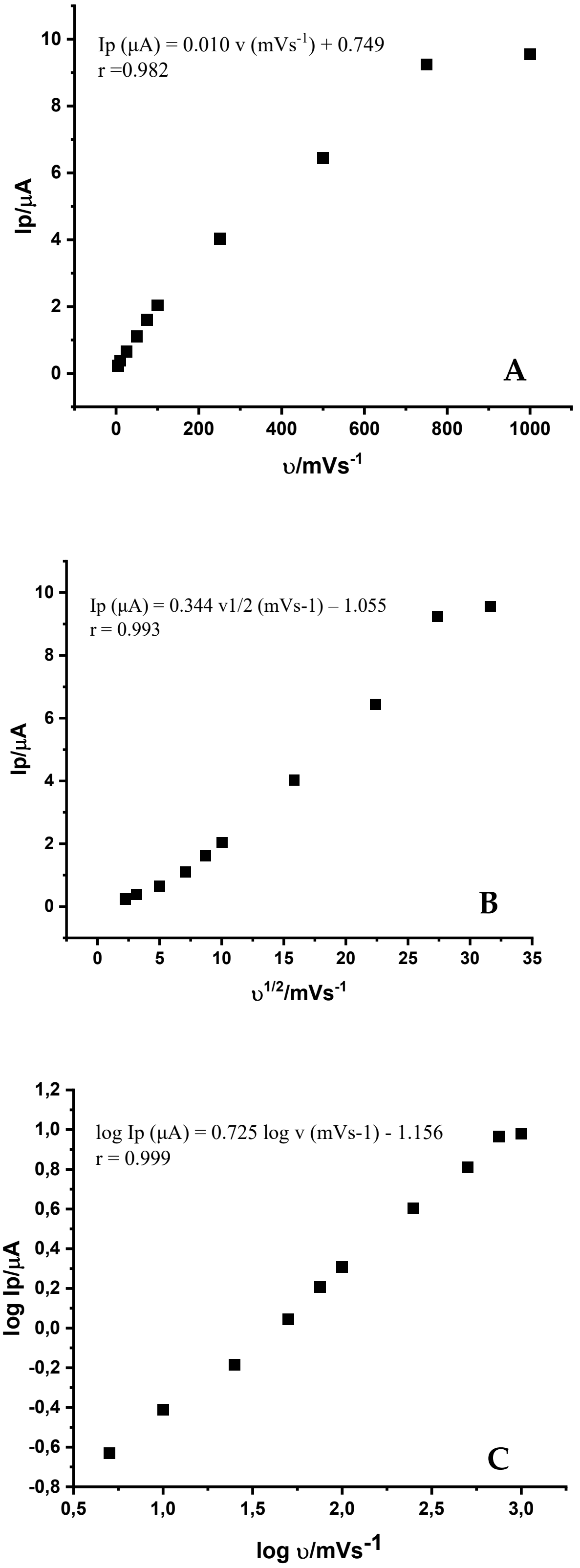

Figure S2. The graphs of 1 × 10^{-4} M CFT solutions in 0.1 M H_2SO_4 by nanosensor (**A**) ip- υ (**B**) ip-$υ^{1/2}$ (**C**) log υ-log ip
